# Characteristics of Mitochondrial Transformation into Human Cells

**DOI:** 10.1038/srep26057

**Published:** 2016-05-17

**Authors:** E. E. Kesner, A. Saada-Reich, H. Lorberboum-Galski

**Affiliations:** 1 Department of Biochemistry and Molecular Biology, Institute for Medical Research Israel-Canada (IMRIC), Faculty of Medicine, Hebrew University of Jerusalem, Jerusalem, 91120, Israel; 2 Monique and Jacques Roboh Department of Genetic Research, Department of Genetics and Metabolic Diseases, Hadassah, Hebrew University Medical Center, Jerusalem, Israel

## Abstract

Mitochondria can be incorporated into mammalian cells by simple co-incubation of isolated mitochondria with cells, without the need of transfection reagents or any other type of intervention. This phenomenon was termed mitochondrial transformation, and although it was discovered in 1982, currently little is known regarding its mechanism(s). Here we demonstrate that mitochondria can be transformed into recipient cells very quickly, and co-localize with endogenous mitochondria. The isolated mitochondria interact directly with cells, which engulf the mitochondria with cellular extensions in a way, which may suggest the involvement of macropinocytosis or macropinocytosis-like mechanisms in mitochondrial transformation. Indeed, macropinocytosis inhibitors but not clathrin-mediated endocytosis inhibition-treatments, blocks mitochondria transformation. The integrity of the mitochondrial outer membrane and its proteins is essential for the transformation of the mitochondria into cells; cells can distinguish mitochondria from similar particles and transform only intact mitochondria. Mitochondrial transformation is blocked in the presence of the heparan sulfate molecules pentosan polysulfate and heparin, which indicate crucial involvement of cellular heparan sulfate proteoglycans in the mitochondrial transformation process.

Mitochondria are critical for the normal function of cells and besides their indispensable role in ATP production, they also take a part in apoptosis, iron metabolism^1^,^2^,^3^, calcium homeostasis^4^,^5^, heme synthesis^6^, steroid biosynthesis^7^,^8^ and more. Numerous diseases and disorders are associated with mitochondrial dysfunctions and mutations, including metabolic pathologies^9^,^10^,^11^,^12^,^13^ and neurodegenerative diseases^14^,^15^,^16^.

It was first reported three decades ago, in 1982, that isolated mitochondria can be incorporated *in vitro* into cells by a simple co-incubation of isolated mitochondria with cells, without the need for transfection reagents, supplements to the medium or any other type of intervention^17^. Originally, this process was named “mitochondrial transformation”. The transformed mitochondria are functional inside the recipient cells, as they can increase ATP production, oxygen consumption and proliferation in rho zero cells and other types of cells, and can replace depleted mitochondrial DNA (mtDNA) in rho zero cells^18^,^19^,^20^,^21^. Moreover, *in vivo*, injection of isolated mitochondria into the heart of rabbits can protect the heart from ischemia-reperfusion injury and increase post-ischemic functional recovery^22^.

Surprisingly, the process of this intriguing phenomenon has not been widely investigated, and there is little data regarding its mechanism(s). Several reports demonstrated that mitochondrial transformation is temperature-, time- and dose-dependent^18^,^19^,^20^,^21^. Other publications examined the mechanisms of mitochondrial transformation. One study in cardiomyocytes found that the transformation was unaffected by pharmacological inhibitors of macropinocytosis, caveolae-dependent-clathrin dependent endocytosis or tunneling nanotubes formation but was blocked by inhibition of actin polymerization^18^. However, other studies demonstrated that pharmacological inhibition of macropinocytosis and caveolae-dependent endocytosis impeded mitochondrial transformation in uterine endometrial cancer cells and Colo205 cells, respectively^19^,^21^.

Here, we report that mitochondrial transformation is, most probably, a biological process and that the transformed mitochondria can co-localize with endogenous mitochondria very rapidly. Transmission electron microscopy (TEM) revealed that the isolated mitochondria interact directly with the recipient cells, which engulf the mitochondria with cellular extensions that might suggest mitochondrial transformation occurs via macropinocytosis. Indeed, macropinocytosis inhibitors but not clathrin-mediated endocytosis inhibition treatments, blocks mitochondria transformation. We also show that pentosan polysulfate and heparin, both heparan sulfated polysaccharide molecules, compete with the mitochondria for interaction with the recipient cells and block the transformation. In addition, damage to the integrity of the mitochondrial outer membrane or to the mitochondrial outer membrane proteins, decreases mitochondrial transformation in a time- and dose-dependent manner, suggesting that cells can distinguish mitochondria from similar particles formed by these treatments.

## Results

### Transformation of isolated mitochondria into cultured cells

We first prepared cells harboring a stable transfection with the plasmid DsRed2-Mito. This plasmid encodes fusion of Discosoma sp. red fluorescent protein and the mitochondrial targeting sequence (MTS) from subunit VIII of human cytochrome c oxidase, which targets the protein to the mitochondria (Fig. S1A).

**Figure 1:**
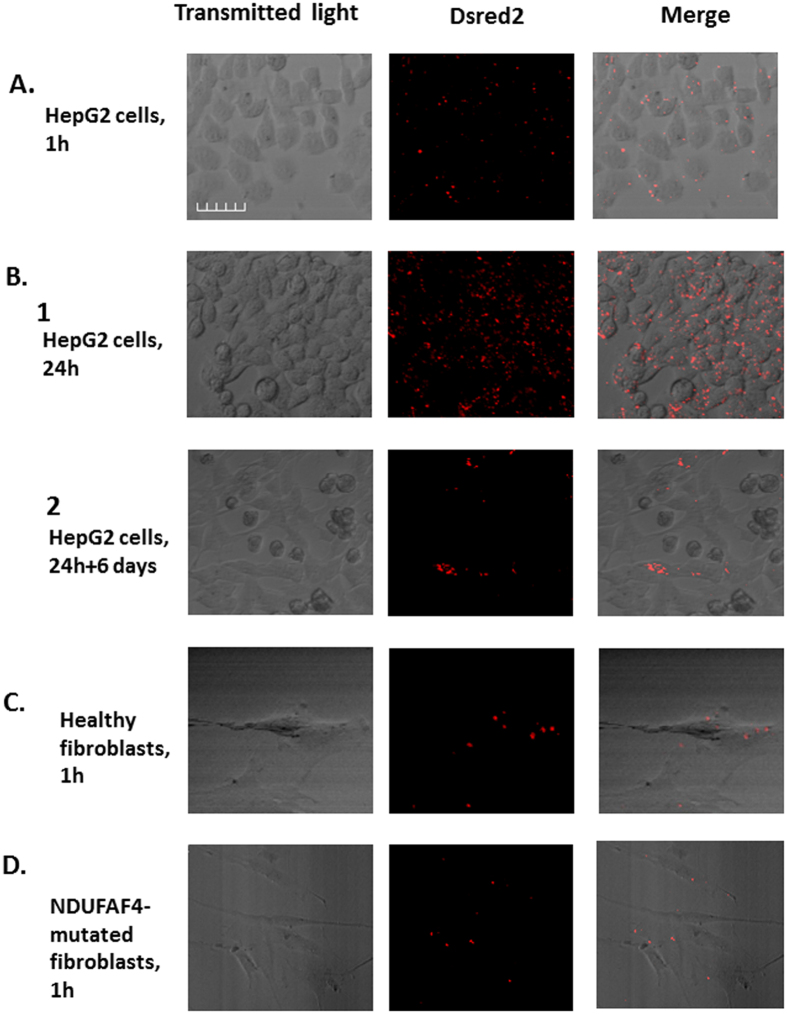
Transformation of isolated mitochondria into cells. Mitochondria were isolated from HeLa-DsRed2-mito cells and incubated (300 μg mitochondria/ml) for 1 h or 24 h with HepG2 cells, healthy fibroblasts, or fibroblasts taken from a patient with a mutation in the gene C6orf66 (NDUFAF4), which encodes the NDUFAF4 assembly factor of complex I. Following the incubation the cells were washed 3 times with PBS, and the medium was replaced with fresh medium. In cases when cells were grown for more than 24 h following the incubation, the cells were divided daily. (**A**) HepG2 cells, following 1 h incubation; (**B1**): HepG2 cells, following 24 h incubation; (**B2**): HepG2 cells, 6 days following 24 h incubation; (**C**) healthy fibroblasts, following 1 h incubation; (**D**) NDUFAF4-mutated fibroblasts, following 1 h incubation. Images were taken using a confocal microscope (Olympus FV1000, X20). Scale bar: 50 μm.

Mitochondria were isolated from these cells, added to human MCF7 cells (breast cancer carcinoma), HEK 293 cells (embryonic kidney), and HepG2 cells (hepatocellular carcinoma), and incubated for 1 h or 24 h. Images were taken using a confocal microscope, at 1 h, 24 h and 6 days following the incubation. Our results show that the isolated mitochondria were transformed into the HepG2 cells (Fig. 1A,B), MCF7 cells and HEK 293 cells (Fig. S2), and that transformation persisted for at least 6 days in HepG2 cells following 24 h incubation (Figs 1B1 and 2).

**Figure 2:**
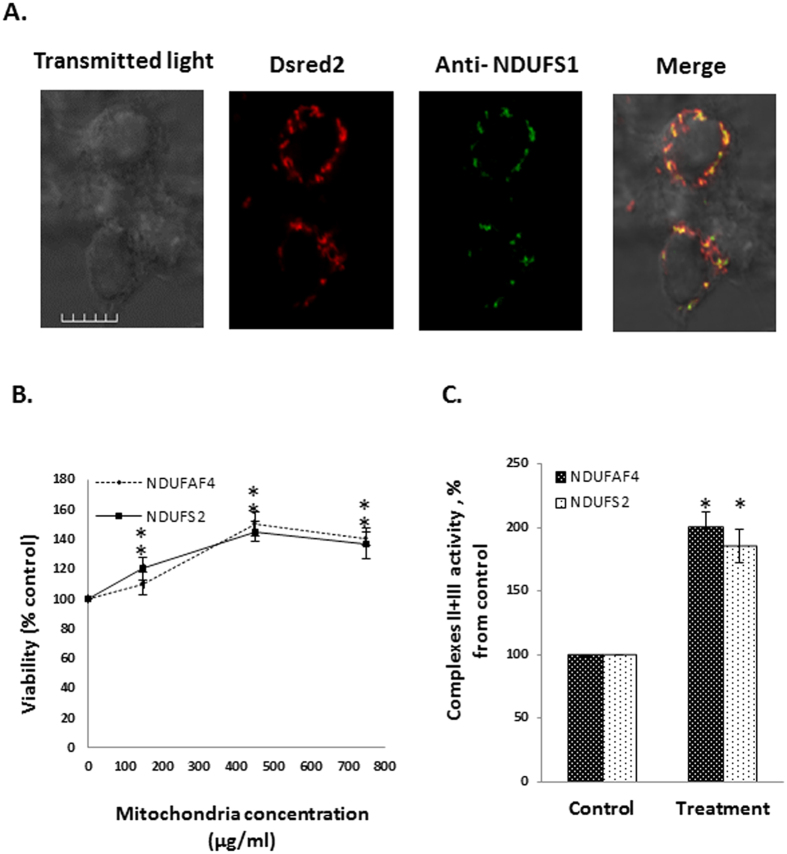
Transformed mitochondria identity confirmation and function within recipient cells. (**A**) Confirmation of transformed mitochondria identity. Mitochondria were isolated from HeLa-DsRed2-mito cells and incubated (300 μg mitochondria/ml) for 1 h with HepG2 cells. Following the incubation the cells were washed 3 times with PBS, and the cells were subjected to immunofluorescence staining with anti-NDUFS1 antibody and secondary 488-dye-conjugated antibody. Red-Dsred2. Green – 488 dye. Scale bar: 50 μm. (**B**) Mitochondrial transformation increases viability of recipient cells. Mitochondria were isolated from HeLa-DsRed2-mito cells and added to NDUFAF4-mutated fibroblasts and NDUFS2-mutated fibroblasts, at the indicated concentrations for 3 days. The cells were then washed 3 times with PBS and cell viability was tested. For control, mitochondria isolation buffer was used. Error bars represent ± SEM, n = 3, ^*^P < 0.005. (**C**) Mitochondria were isolated from HeLa-DsRed2-mito cells, added to NDUFAF4-mutated fibroblasts and NDUFS2-mutated fibroblasts (450 μg/ml) for 3 days, followed by 3 washes with PBS. The activity of complexes II + III was measured. ^*^P < 0.005. Error bars represent ± SEM, n = 3.

To confirm the identity of the transformed mitochondria, the mitochondria-transformed cells were immunostained with anti-NDUFS1 antibody and we found that the red staining (originated form our isolated mitochondria) is co-localized with the green staining (originated form the anti-NDUFS1 antibody), as evidenced by the merged yellow staining (Fig. 2A).

To verify purity of mitochondria isolation, cytoplasmic and mitochondrial sub-fractions were subjected to western blot analysis, using anti-α tubulin (cytoplasm) and anti-pyruvate dehydrogenase E1-a antibodies (mitochondria; Fig. S3A). Completeness and viability of the mitochondria were also verified by measuring citrate synthase activity. Activity of the enzyme was measured only when the isolated mitochondria were pre-treated with tritonX100 (Fig. S3B). Citrate synthase is a matrix mitochondrial enzyme, and substrates added to the mitochondria are not accessible to the enzyme without a detergent (tritonX100), in case mitochondria are intact; thus, this assay reflects both mitochondria integrity and mitochondria viability.

Cancer cells differ from normal cells in many aspects of metabolism and cell biology, and therefore we set out to examine mitochondrial transformation into non-cancerous cells. To this end, we used as recipient cells healthy fibroblasts, fibroblasts taken from a patient with a mutation in the gene C6orf66, which encodes the NDUFAF4 protein, an assembly factor of complex I of the respiratory chain, and G229C/Y35X cells, fibroblasts taken from a patient, with the G229C/Y35X mutation which manifests neonatal period with recurrent liver failure and severe neurodegenerative disease from early infancy^23^. Our results demonstrate that fluorescent-labeled mitochondria are also transformed into healthy fibroblasts (Fig. 1C), NDUFAF4-mutated fibroblasts (Fig. 1D), and G229C/Y35X fibroblasts (Fig. S2), and that the transformation persisted for at least 6 days in G229C/Y35X cells following 24 h incubation (Fig. S2).

These results suggest that isolated mitochondria are transformed into cancer cells, healthy fibroblasts and mitochondrial-mutated cells, just by co-incubation of the cells with isolated mitochondria, and are persistent in the recipient cells for days.

### Mitochondrial transformation can benefits the recipient cells

Next, isolated DsRed2-mito mitochondria were incubated with NDUFAF4-mutated patient’s cells and with fibroblasts of a patient with a mutation in the NDUFS2 gene, which encodes NADH dehydrogenase (ubiquinone) Fe-S protein 2, a core subunit of complex I of the respiratory chain and cell viability was measured. We found that cell viability of both of the patients’ cells was improved in a dose dependent manner (Fig. 2B). We then measured directly the mitochondrial activity of the mitochondrial-transformed cells, and found that following mitochondrial transformation complex II + III activity was increased by ~100 (Fig. 2C) in both NDUFAF4-mutated and NDUFS2-mutated cells. These results indicate that mitochondrial transformation benefits the recipient cells causing an increase in both cell viability and direct mitochondrial-activity.

### Exogenous mitochondria are transformed to recipient cells rapidly and co-localize with endogenous mitochondria

**Figure 3:**
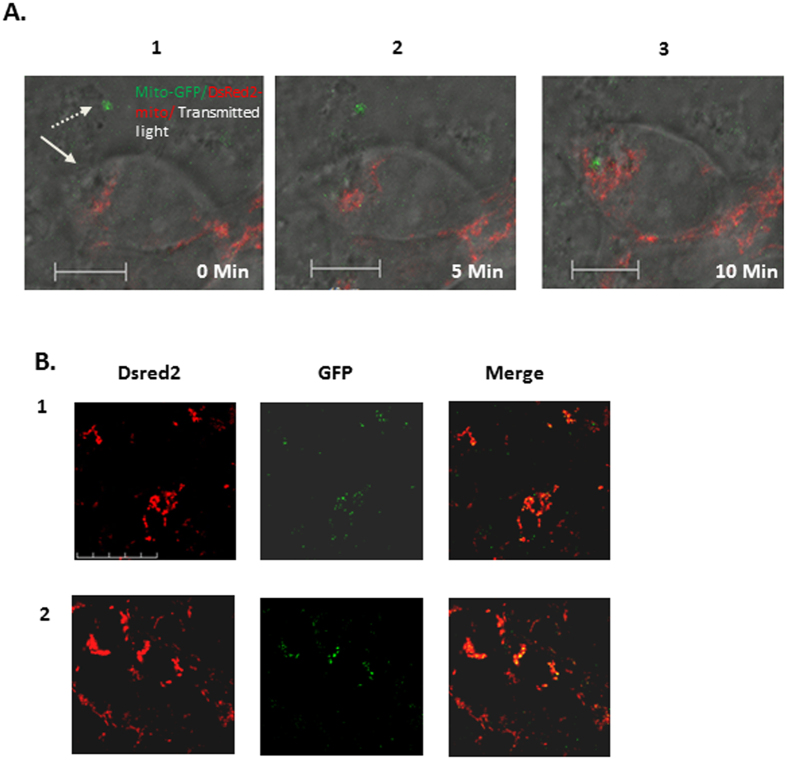
Mitochondrial-transformation’s kinetics and co-localization with the endogenous mitochondria. (**A**) Mitochondria were isolated from HEK293-mito-GFP cells and were added (300 μg/ml) to HeLa-DsRed2-mito cells, followed immediately by live-confocal microscopy recording (ZEISS LSM710, X63). Pictures were taken every 5 min. A1: GFP-labeled mitochondrion near HeLa-DsRed2-mito, at time zero, the time point in which GFP-labeled mitochondrion (marked by dashed arrow) was captured in proximity to HeLa-DsRed2-mito cell (marked by arrow); A2: + 5 minutes; A3: + 10 minutes. Scale bar: 5 μm (**B**) HeLa-DsRed2-mito cells were plated on cover-slips, and 24 h later mitochondria were isolated from Hek293-mito-GFP and added to HeLa-DsRed2-mito cells (300 μg/ml). Following 6 h incubation, excess mitochondria were washed 3 times with PBS, and the medium was replaced with fresh medium. B1-2: two microscope-fields. Images were taken using confocal microscope (Olympus FV1000), X60. Red – Dsred2; green – GFP. Scale bar: 50 μm.

Next, we investigated mitochondrial transformation using live microscopy. HEK 293 cells were transfected with the plasmid pCMV/myc/mito/GFP, which encodes a fusion of the GFP fluorescent protein with the mitochondrial targeting sequence of human cytochrome c oxidase (Fig. S1B). Mitochondria were isolated from HEK293-mito-GFP cells and incubated with HeLa-DsRed2-mito cells, and the transformation was recorded by live microscopy. As can be seen in Fig. 3A1, 2, 3, the kinetics of the transformation are quite fast: only 10 min after a green-labeled exogenous mitochondrion was seen nearby mitochondria-red-labeled cell (Fig. 3A1, 2), it was detected within the recipient cells (Fig. 3A3). Moreover, within the recipient cell, the proximity of the green-labeled exogenous mitochondrion to the red-labeled endogenous mitochondria (Fig. 3A3) suggests co-localization of the exogenous mitochondrion with endogenous mitochondria.

In order to directly explore whether exogenous mitochondria co-localize with the endogenous mitochondria, we examined fixed cells using confocal microscopy. Mitochondria were isolated from HEK293-mito-GFP cells and incubated with HeLa-DsRed2-mito cells, cells were then fixed and examined by confocal microscopy. As can be seen in Fig. 3B1, 2, notable amounts of green-labeled exogenous mitochondria (green fluorescence) co-localized with the red-labeled endogenous mitochondria (red fluorescence), as evidenced by the yellow merge florescence and quantification of the data (Table S1).

### Exogenous mitochondria interact directly with the recipient cells, which engulf the mitochondria

**Figure 4:**
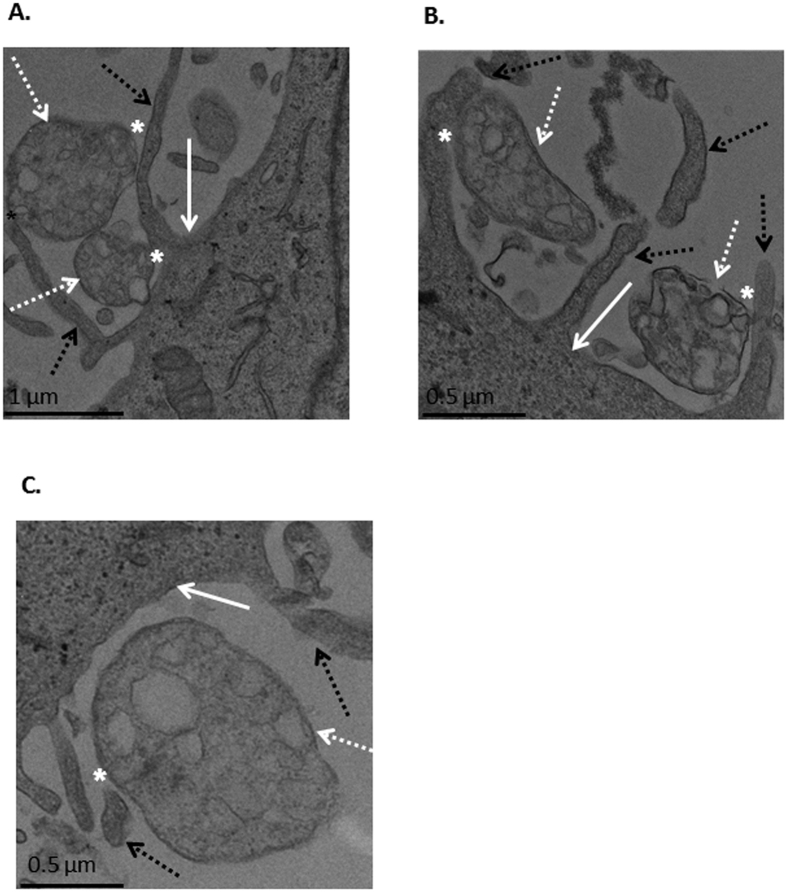
TEM microscopy of mitochondrial interaction with recipient cells. (**A–C**) Mitochondria were isolated from HeLa-DsRed2-mito cells and were incubated with HepG2 cells (300 μg/ml) for 1 h. Following incubation, excess mitochondria were removed by three washes with PBS, and the cells were examined by TEM. Pictures were taken using a Jeol 1400 Plus TEM with an Orius Gatan CCD camera and Gatan digital micrograph program (**A** x6000 magnification; **B** x10,000 magnification; **C** x5000 magnification). White arrow: HepG2 cells; white dashed arrow: exogenous mitochondria; black dashed arrow: cellular excitations; asterisk: contact site.

Cellular debris can also interact with cells and be incorporated into them^24^,^25^,^26^,^27^,^28^, and therefore it was possible that cell remnants and detritus present in the mitochondrial fraction interacted in a non-specific manner with the mitochondria and the cells and mediated the transformation of the mitochondria into the cells. To examine the mitochondrial interaction with recipient cells, we used TEM analysis. Mitochondria isolated from HeLa DsRed2-mito cells were added to HepG2 cells, and following 1 h incubation the cells were fixed and subjected to TEM microscopy. As demonstrated in Fig. 4A–C and S4A–D, mitochondria interacted directly with the cells. It can also be seen that the recipient cells engulfed the mitochondria by cellular extensions and cell surface ruffling, which might indicate macropinocytosis involvement^29^,^30^. It should be pointed out that some of the exogenous mitochondria are irregularly shaped, probably due to the isolation procedure and the presence of calcium in the incubation medium; however, the shape of our mitochondria is in line with previous reports done with isolated mitochondria^19^.

### Macropinocytosis inhibition, but not clathrin-mediated endocytosis inhibition, blocks mitochondria transformation

We investigated whether efficacy of mitochondrial transformation could be assessed by measuring the fluorescence of DsRed2-labeled mitochondria-transformed cells, and found that indeed the transformation can be examined and quantified in this manner (Fig. S5).

**Figure 5:**
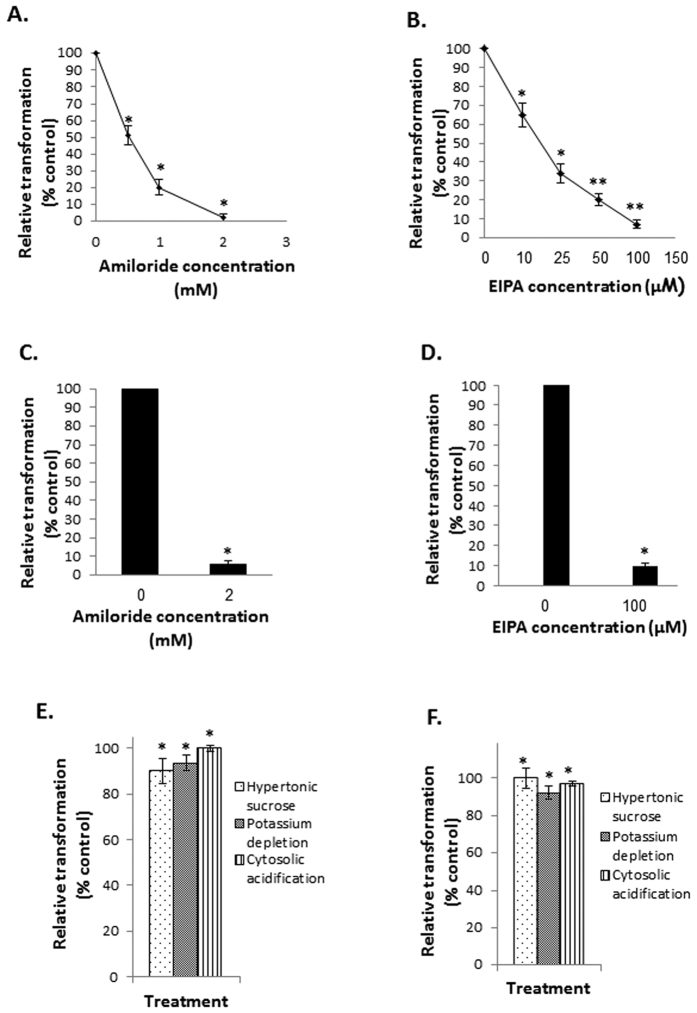
Inhibition of macropinocytosis, but not of clathrin-mediated endocytosis, impede mitochondrial transformation. (**A,C**) Mitochondria were isolated from HeLa-DsRed2-mito cells and were incubated (300 μg/ml) with HepG2 cells (**A**) or healthy fibroblasts (**C**) pre-treated with the macropinocytosis inhibitor amiloride (30 minutes, at indicated concentration) or vehicle (DMSO) for 1 h. Following incubation, excess mitochondria were removed by three washes with PBS, and membrane-bound mitochondria were removed by incubation with heparin (200 μg/ml, 30 minutes) and 3 PBS washes. Fluorescence was recorded in a plate reader and is calculated as percentage from control cells, and represented as relative transformation. Error bars represent SEM, n = 3, ^*^P < 0.05. (**B,D**) Mitochondria were isolated from HeLa-DsRed2-mito cells and were incubated (300 μg/ml) with HepG2 cells (**B**) or healthy fibroblasts (**D**) pre-treated with the macropinocytosis inhibitor EIPA (30 minutes, at indicated concentration) or vehicle (DMSO) for 1 h. Following incubation and washings (as described for **A** and **C**; see above), fluorescence was recorded in a plate reader and is calculated as percentage from control cells, and represented as relative transformation. Error bars represent SEM, n = 3, ^*^P < 0.05, ^**^P < 0.03. (**E,F**) Mitochondria were isolated from HeLa-DsRed2-mito cells and were incubated (300 μg/ml) with HepG2 cells (**E**) or healthy fibroblasts (**F**), pre-treated with the clathrin-mediated endocytosis inhibitory treatments: hypertonic sucrose (0.5 M, 30 minutes), potassium depletion (as described in materials and methods section) and cytosolic acidification (25 mM ammonium chloride, 30 minutes) for 1 h. Following incubation and washings (as described for A and C; see above), fluorescence was recorded in a plate reader and is calculated as percentage from control cells, and represented as relative transformation. Error bars represent SEM, n = 3, ^*^P < 0.05.

Next, we verified the possible involvement of macropinocytosis in mitochondrial transformation. Mitochondria were isolated from HeLa DsRed2-mito cells, and were added to HepG2 cells pre-treated with amiloride or 5-(N-ethyl-N-isopropyl)-Amiloride (EIPA), common macropinocytosis inhibitors. Our results show that both amiloride and EIPA inhibited the transformation in a dose-deepened manner (Fig. 5A,B). These experiments were repeated with healthy fibroblasts, and showed similar results (Fig. 5C,D). In order to exclude clathrin-mediated endocytosis, mitochondria were isolated from HeLa DsRed2-mito cells and were added to HepG2 cells pre-treated with clathrin-mediated endocytosis inhibition-treatments (hypertonic sucrose, potassium depletion and cytosolic acidification). Inhibition of clathrin-mediated endocytosis didn’t affect mitochondrial transformation (Fig. 5E). These experiments were repeated with healthy fibroblasts, and showed similar results (Fig. 5F). Thus, mitochondria uptake by recipient human cells most probably involves macropinocytosis mechanisms.

### Integrity of the mitochondrial outer membrane is essential for its transformation into cells

**Figure 6:**
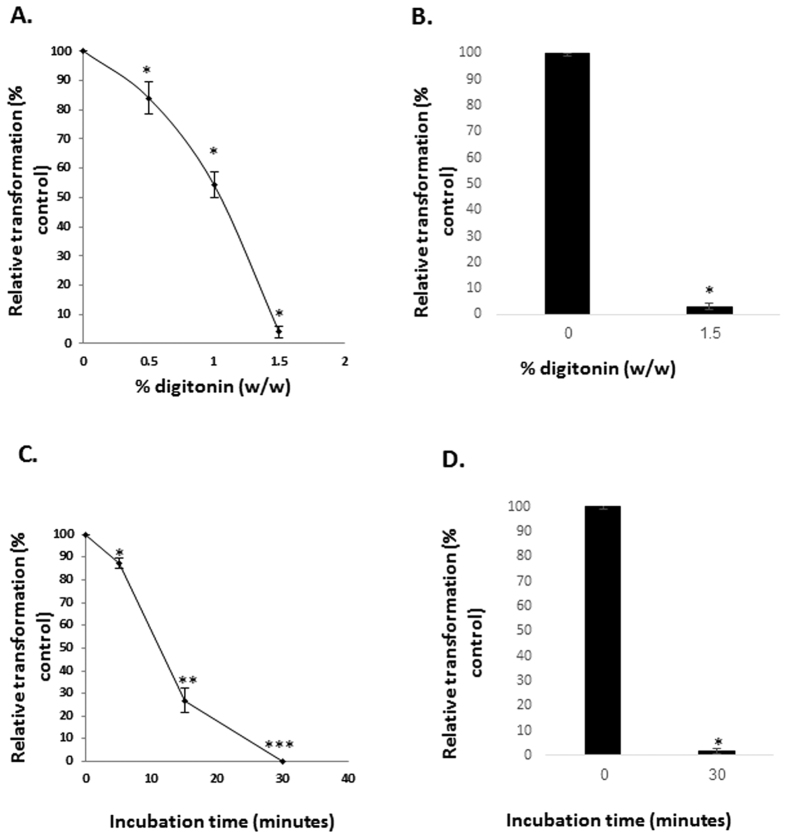
Mitochondria outer membrane integrity is essential for mitochondrial- transformation. (**A,B**) Effect of digitonin treatment on mitochondrial transformation. Mitochondria were isolated from HeLa-Dsred2-mito cells and were treated with digitonin or DMSO (control, 0) at the indicated concentrations (% from mitochondria, w/w) for 10 min on ice. Following three washes, the mitochondria (300 μg/ml) were added to (**A**) HepG2 cells or (**B**) healthy fibroblasts, plated in 96 well plates, and incubated for 1 h. Excess mitochondria were removed by three washes with PBS, and fluorescence intensity was recorded. Error bars represent ± SEM, n = 3, ^*^P < 0.005. (**C,D**) Effect of swelling treatment on mitochondrial transformation. Mitochondria were isolated from HeLa-DsRed2-mito cells, and were treated with swelling buffer (20 mM HEPES, 90% v/v) or isolation buffer (control, 0) for the indicated time periods. Following three PBS washes, the mitochondria (300 μg/ml) were added to (**C**) HepG2 cells or (**D**) healthy fibroblasts, plated in 96 well plates and incubated for 1 h. Excess mitochondria were removed by three washes with PBS, and fluorescence intensity was recorded. The florescence of each treatment is calculated as percentage from control cells, and represented as relative transformation. In all the experiments, the fluorescence completeness of the treated-isolated mitochondria was recorded, and found to be equal to un-treated mitochondria. Error bars represent ± SEM, n = 3, ^*^P < 0.03; ^**^P < 0.006; ^***^P < 0.0002.

We next tested the specificity of transformation for intact mitochondria. Mitochondria were treated with digitonin, which harms the outer mitochondrial membrane, but not the inner membrane. This treatment results in a particle of similar size to a mitochondrion, but with a different surface. HeLa-DsRed2-mito isolated mitochondria were treated with increasing concentrations of digitonin, incubated for 1 h with HepG2 cells and the fluorescence of the cells was recorded. We found that the digitonin treatment decreased mitochondrial transformation in a dose-dependent manner, and that at high concentrations it completely abolished the transformation (Fig. 6A). Digitonin treatment alone did not affect the fluorescence of the mitochondria itself (data not shown). Experiments performed with healthy fibroblast showed similar results (Fig. 6B).

Another way to damage the mitochondrial outer membrane is by incubating with swelling buffer, which disrupts the outer mitochondrial membrane, while having no effect on the inner membrane. This treatment was conducted for increasing periods of time, and we found that it decreased mitochondrial transformation in a time-dependent manner and could totally abolish the transformation (Fig. 6C). The swelling treatment in itself did not affect mitochondrial fluorescence (data not shown). These experiments were also performed with healthy fibroblasts, and showed similar results (Fig. 6D).

These results indicate that mitochondrial outer membrane integrity is essential for its transformation into cells, and that a similar particle, with a different surface, cannot be transformed into recipient cells.

### Mitochondrial transformation requires interaction with cellular heparan sulfate proteoglycan

**Figure 7:**
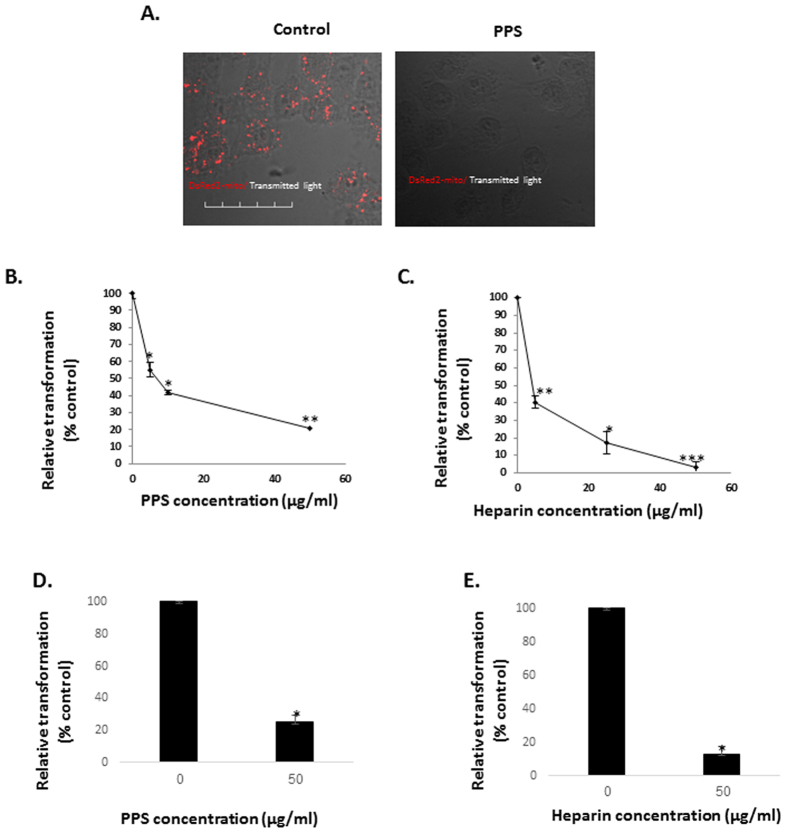
Heparan sulfated polysaccharide molecules are essential for mitochondrial-transformation. (**A**) Microscopy. Mitochondria isolated from HeLa-DsRed2-mito were added (300 μg/ml) to HepG2 cells, pre-treated (2 min) with PPS (200 μg/ml) or vehicle. Following 1 h incubation, excess mitochondria were removed by three washes with PBS, and the cells were examined by confocal microscopy (Olympus FV-1000; x 60) Scale bar: 50 μm. (**B–E**) Fluoresce recording testing. Mitochondria were isolated from HeLa-DsRed2-mito cells and added (300 μg/ml) to HepG2 cells, plated in 96-well plates, pre-treated (2 min) with (**B**) PPS or (**C**) heparin at the indicated concentrations, or to healthy fibroblasts, plated in 96-well plates pre-treated (2 min) with (**D**) PPS or (**E**) heparin at the indicated concentrations, or vehicle. Following 1 h incubation, the cells were washed three times in PBS to remove excess mitochondria, and the fluorescence was recorded in a plate reader. The florescence of each treatment is calculated as percentage from control cells, and represented as relative transformation. Error bars represent ± SEM, n = 3. PPS: ^*^P < 0.005, ^**^P < 0.004 ^**^P < 0.0003. Heparin: ^*^P < 0.007; ^**^P < 0.004; ^***^P < 0.002.

As many cell interactions require the involvement of heparan sulfate, we investigated its involvement in mitochondrial transformation. Incubation of recipient cells with mitochondria isolated from HeLa-Ds-Red2-mito cells in the presence of pentosan polysulfate (PPS), a glycosaminoglycan which is commonly used in order to compete with heparan sulfate-dependent interactions, abolished mitochondrial transformation in a dose-dependent manner (Fig. 7A,B). Another polysaccharide that is used for to compete with polysaccharide-dependent interactions is heparin. We tested mitochondrial transformation in the presence of heparin, and found that similar to PPS, heparin also inhibited mitochondrial transformation in a dose-dependent manner (Fig. 7C). PPS and heparin also inhibited mitochondrial transformation into healthy fibroblasts (Fig. 7D,E).

### Integrity of the mitochondria outer membrane proteins is essential for its transformation into cells

**Figure 8:**
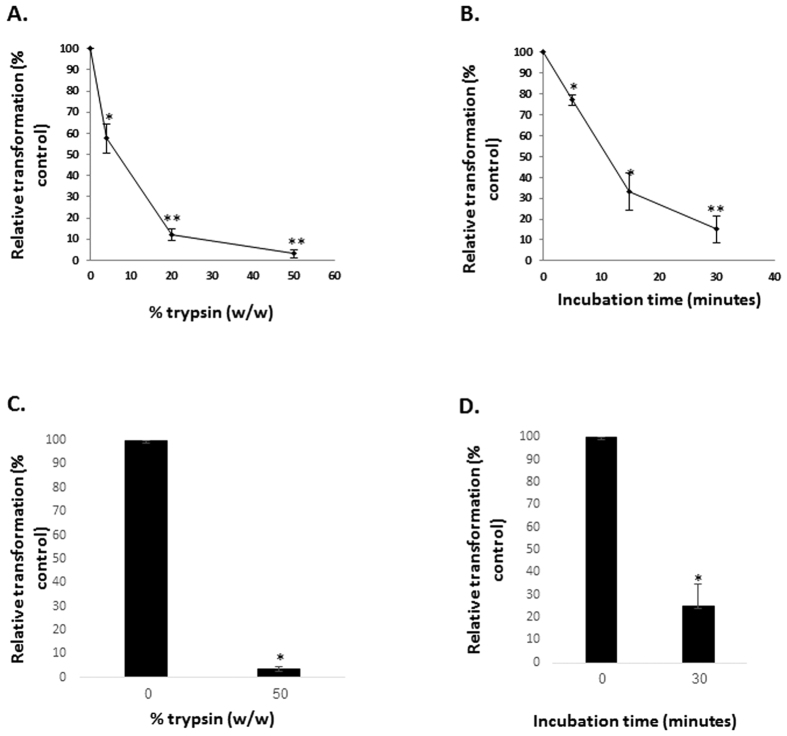
Mitochondrial outer membrane proteins integrity is essential for mitochondria transformation. (**A,C**) Mitochondria were isolated from HeLa-DsRed2-mito cells and treated with trypsin at the indicated concentration (% from mitochondria, w/w) for 10 min on ice. For control, 20% (v/v) FBS was added in addition to trypsin. Following three washes, the mitochondria (300 μg/ml) were added to (**A**) HepG2 cells or (**C**) healthy fibroblasts, plated in 96 wells plates, and were incubated for 1 h. The cells were washed three times in PBS to remove excess mitochondria, and the fluorescence was recorded in a plate reader. Error bars represent ± SEM, n = 3, ^*^P < 0.004; ^**^P < 0.0002. (**B,D**) Mitochondria were isolated from HeLa-DsRed2-mito cells and treated with trypsin at a concentration of 2% (w/w), at the indicated time periods on ice. For control, 20% (v/v) FBS was added in addition to trypsin. Following three washes, the mitochondria (300 μg/ml) were added to (**B**) HepG2 cells or (**D**) healthy fibroblasts, plated in 96 wells plates, and were incubated for 1 h. The cells were washed three times in PBS to remove excess mitochondria, and the fluorescence was recorded in a plate reader. The florescence of each treatment was calculated as percentage from control cells, and represented as relative transformation. In all the experiments, the fluorescence of the mitochondria alone was tested in a plate reader and was found equal to untreated mitochondria. Error bars represent SEM, n = 3, ^*^P < 0.02; ^**^P < 0.006.

Next, we wanted to test whether harming the mitochondrial outer membrane proteins would influence mitochondrial transformation into cells. We hypothesized that the outcome of such treatment would be particles of similar size and with a similar fatty acid surface composition to that of intact mitochondria, but with a different protein surface content. HeLa-Ds-Red2-mito mitochondria were treated with increasing concentrations of trypsin or at a constant concentration for increasing periods of time, and incubated for 1 h with HepG2 cells. Trypsin treatment decreased mitochondrial transformation in a dose- (Fig. 8A) and time-dependent (Fig. 8B) manner. The trypsin treatments did not affect the mitochondrial fluorescence itself. Experiments performed with healthy fibroblast showed similar results (Fig. 8C,D). These results suggest that mitochondrial outer membrane proteins function, most probably, as ligands for mitochondrion-cell interaction, and harming these proteins results in failure to conduct mitochondrion-cell interactions and thus mitochondrial transformation.

## Discussion

Although mitochondrial transformation of isolated mitochondria into host cells was reported over 30 years ago and has been well established since then, there is still not much knowledge about the mechanism(s) of this phenomenon. While mitochondrial transformation’s beneficial effects are clear, the process itself is still vague.

In this research we studied some mechanistic characteristics of mitochondrial transformation. We showed that mitochondria can be easily transformed into cancer cells, healthy fibroblast and fibroblasts culture from patients with mitochondrial deficiencies, and that they improve cell viability and mitochondrial activity in the defective cells. Transformed mitochondria can also co-localize with endogenous mitochondria very rapidly (Figs 1,2,3A and S2). Transmission electron microscopy (TEM) revealed that the isolated mitochondria interact directly with the recipient cells, which engulf the mitochondria with cellular extensions that might suggest mitochondrial transformation occurs via macropinocytosis. Indeed, macropinocytosis inhibitors but not clathrin-mediated endocytosis inhibition, blocks mitochondria transformation. (Figs 3 and 4). Importantly, digitonin and swelling treatments (Fig. 6), which disrupt the mitochondrial outer membrane, and trypsin treatment (Fig. 8), which damages the mitochondrial outer membrane proteins, blocked mitochondrial transformation in a time- and dose-dependent manner in HepG2 cells and in healthy fibroblasts. We also demonstrated, in both healthy fibroblasts and HepG2 cells cultures, that the polysaccharides PPS and heparin abolished mitochondrial transformation (Fig. 7).

Cells can incorporate cell debris and artificial nano-particles can also be imported to cells^24^,^25^,^26^,^27^,^28^,^31^,^32^,^33^,^34^. From this perspective, the ability of cells to differentiate intact mitochondria from digitonin, swelling and trypsin-treated mitochondria, as demonstrated in our study (Figs 6 and 8) is notable; the cells ignore these semi-mitochondrial particles, and recognize and transform only intact mitochondria. Interestingly, some viruses evolved to camouflage themselves as cell debris in order to be incorporated into cells by endocytosis^30^,^35^, whereas mitochondria, according to our data, is transformed into cells only when it is distinguished from cell debris. In addition, when cells consume cells debris they often swell and their shape gets abnormal and irregular, but this was not seen in mitochondrial transformation, not by us and not by others^17^,^18^,^19^,^21^,^22^. These results suggest that mitochondrial transformation utilizes different process(es) from cell debris uptake and is, most probably, a biological phenomenon.

The inability of the trypsin-treated mitochondria, which form particles similar to mitochondria but different in their surface proteins, to be transformed into cells, may imply that specific protein or proteins, located in the mitochondrial outer membrane, mediates mitochondrial transformation into cells. These proteins may function as ligands in the cell-mitochondria interactions. There are specific mitochondrial outer membrane proteins that take a part in interactions of mitochondria within the cells with other intracellular compartments or with the mitochondrial network^36^,^37^,^38^,^39^,^40^,^41^,^42^,^43^,^44^, and it is feasible that specific proteins (perhaps some of the same proteins which participate the intracellular interactions) may play a role in the interactions of mitochondria with the cell outer membrane. On the other hand, specific cell membrane proteins may also be required for the mitochondria-cell interaction, thus, may function as receptor(s) for the mitochondria-cell interactions.

There are evidences for the involvement of at least three mechanisms in mitochondrial transformation: actin-dependent endocytosis, macropinocytosis and caveolae-dependent endocytosis^18^,^19^,^21^; however, reports regarding the mechanism(s) of mitochondrial transformation are inconsistent. In this regard, it should be pointed out that the reported studies used different cells types, protocols and conditions.

We tested mitochondrial transformation into HepG2 cells, as well as into healthy fibroblasts. TEM experiments with HepG2 cells show that the cells engulf the mitochondria with cellular extensions (Fig. 4) in a way that suggests the involvement of macropinocytosis or macropinocytosis-like mechanisms in mitochondrial transformation into human cells. Clathrin-mediate endocytosis, caveolae-dependent endocytosis, lipid raft and other endocytosis mechanisms don’t involve the interaction with cellular extensions^29^,^30^. In agreement with these observations, pharmacological tests revealed that macropinocytosis inhibitors, but not caveolae-dependent endocytosis inhibitor treatments, blocked mitochondrial transformation (Fig. 5).

We have shown that mitochondrial transformation is blocked by heparan sulfate molecules; pentosan polysulfate and heparin, which indicate crucial involvement of cellular heparan sulfate proteoglycans in the mitochondrial transformation process. Interestingly, cellular heparan sulfate proteoglycans are involved in incorporation of viruses and bacteria into cells^45^,^46^. Unlike mitochondrial transformation, these processes were widely studied, and our results might suggest that mechanisms of viruses and bacteria entry into cells might be useful to explore mitochondrial transformation.

A different but similar procedure to mitochondrial transformation is the transfer of mitochondria from cell to cell by direct cell-to-cell nanotube channels. An *in vivo* study reported that bone marrow derived stromal cells can protect against acute lung injury induced by LPS, and that this protection is based on mitochondrial transfer between the stromal cells to the damaged cells by connexin-containing gap junctional channels^47^. Despite the fact that the involvement of nanotube channels in mitochondrial transformation was rejected in one study^18^ and was not seen in another^22^, it is possible that the two phenomena share some mechanisms and pathways. In addition, the encouraging outcomes of mitochondrial transfer between cells by connexin-containing gap junctional channels increases the possibility that mitochondrial transformation could also be used for therapeutic use.

The capacity of mitochondria to be transformed into mitochondria-deficient patient cells (Fig. 1C,D) together with our finding (Fig. 2B,C) and that of others (see above) about the capability of exogenous mitochondria to improve mitochondrial biochemical functions of mitochondria-defective cells, might suggest a potential therapy for genetic mitochondrial disorders. As for nuclear-encoded mitochondrial diseases, mitochondrial transformation may offer a potential new approach for therapy, nevertheless, depending on the half-life of mitochondrial proteins or its complexes, this therapy will have to be, most probably, a chronic treatment.

However, the potential for mtDNA-encoded pathologies is even greater. Mitochondrial DNA mutations cause disease in >1 in 5000 of the population, and approximately 1 in 200 of the population are asymptomatic carriers of a pathogenic mtDNA mutation^48^. The mtDNA mutation disorders can provoke a variety of clinical pathologies, including blindness, deafness, muscle myopathies and death, which can appear at any age. Despite the urgent need to develop treatments for these diseases and the substantial efforts made in the field, there is currently a lack of satisfying remedies for these illnesses^49^. Mitochondrial transformation based therapy could offer a potential treatment for many of these disorders, as it has the ability to improve mitochondrial dysfunctions in diverse conditions. Moreover, transformed mitochondria can replace depleted mitochondrial mtDNA in rho zero cells^18^, and therefore it is possible that mitochondrial transformation based therapy will result in exchanging of the mutated mtDNA with normal mtDNA and thus will promote permanent, full or partial, reinstatement of mitochondrial activity, and spare the need for chronic treatment.

## Materials and Methods

### Cells

HepG2, HeLa, HEK-293 and MCF7 cells were obtained from ATCC (Manassas, VA). Primary fibroblast cultures from patients and healthy donors were obtained from forearm skin biopsies (with informed consent). One of the patients carried a homozygous mutation in the gene C6orf66 (NDUFAF4) with a T/C substitution at nucleotide 194 that predicts aLeu65Pro mutation. Another patient carried a homozygous Arg228Gln mutation in the gene NDUFS2. The third patient carries the G229C/Y35X mutation, which manifests neonatal period with recurrent liver failure and severe neurodegenerative disease from early infancy.

All experiments on human primary cultures were performed according to the ethical guidelines of the Hadassah Medical Center (Helsinki committee) and Hebrew University. All subjects provided written informed consent. All experimental protocols were approved by Hadassah Medical Center (Helsinki committee) and the Hebrew University, Department of Safety.

Cell lines and primary fibroblast cultures were grown in Dulbecco’s modified Eagle medium (DMEM), supplemented with 10% fetal calf serum, 1% glutamine and 1% penicillin-streptomycin (Biological Industries, Beit Ha’emek, Israel) at 37 °C in humidified atmosphere of 5% CO_2_.

### Plasmids and transfections

The plasmid DsRed2-Mito (Clontech, Mountain View, CA) encodes fusion of Discosoma sp. red fluorescent protein and the mitochondrial targeting sequence (MTS) from subunit VIII of human cytochrome c oxidase, which targets the protein to the mitochondria. HeLa cells were stably transfected with this plasmid according to manufacturer’s manual (HeLa DsRed2-mito cells, Fig. S1A), and stability was preserved by selection with G418 (Calbiochem, San Diego, CA, 0.6 mg/ml). The plasmid CMV/myc/mito/GFP (Invitrogen, Cambridge, MA) encodes a fusion of GFP fluorescent protein, and the mitochondrial targeting sequence from subunit VIII of human cytochrome c oxidase. HEK-293 cells were stably transfected with this plasmid according to manufacturer’s manual (HEK-293-GFP-mito cells; see supplemental data Fig. S1B), and stability was preserved by selection with G418 (1.2 mg/ml).

### Mitochondrial isolation

Cells were collected by trypsinization, suspended in PBS, and centrifuged (5 min, 250 g) twice. Mitochondrial isolation procedures were performed at 4 °C or on ice and in the dark (to prevent fluorescence decay). The centrifuged cells were re-suspended in mitochondrial isolation buffer (320 mM sucrose, 5 mM Tris-HCl, pH 7.4, 2mM EGTA), and homogenized with a Dounce homogenizer. Nuclei and cell debris were removed by two centrifugations at 3000 g for 5 min and the supernatant was collected. The supernatant was then centrifuged at 12,000 g for 10 min, and the mitochondrial pellet was re-suspended in mitochondrial isolation buffer. Mitochondrial concentration was determined by Bradford assay. All the experiments were performed with freshly isolated mitochondria.

### Bradford assay

Bradford assay was performed using the Bio-Rad protein assay (Bio-Rad, Hercules, CA) according to the manufacturer’s manual.

### Western blot analysis

Total protein extraction was performed by lysing the cells with lysis buffer (20 mM Tris-Hcl, 100 mM NaCl, 1mM EDTA, 0.1% (v/v) SDS, 1 mM PMSF) for 1 min, incubation of the lysate for 5 min at 4 °C, followed by centrifugation at 14,000 g and 4 °C for 10 min, and collection of the supernatant. Isolated mitochondria (see above) were used as the mitochondrial sub-fraction. The supernatant above the mitochondrial pellet was used as the cytosolic sub-fraction. Protein concentration was determined by Bradford assay. Samples (100 μg/lane) were separated on 12% sodium dodecyl sulfate-polyacrylamide electrophoresis (SDS-PAGE) gels, and then the proteins were electro-transferred onto Immobilon-P transfer membrane (Millipore, Bradford, PA). The membrane was then blotted with anti-pyruvate dehydrogenase E1α (Molecular Probes, Eugene, OR), 1:1000, and anti-α-tubulin (Serotec, Oxford, UK) 1:10,000. Visualization of the bands was achieved using an enhanced chemiluminescence kit (ECL; Biological Industries, Beit Ha’emek, Israel).

### Citrate synthase assay

Mitochondria were isolated as described above. Citrate synthase assays were performed in order to validate the integrity of the isolated mitochondria. The assay procedure was carried out as previously described^50^.

### Mitochondrial transformation into cells

A day following cell plating (at 50,000 cells/ml for HepG2 and MCF7 cells; 30,000 cells/ml for HEK-293; 75,000 cells/ml for fibroblasts), mitochondria were added to plated cells at the indicated concentrations. The plate was gently shaken for 30 s, and then returned to the incubator at 37 °C. For heparan sulfate competition assays, pentosan polyphosphate (PPS; Sigma, St. Louis, MO) or heparin (Sigma, St. Louis, MO) were added 2 min before addition of the isolated mitochondria, at the indicated concentrations, followed by gentle shaking for 30 s. Unless stated otherwise, incubations were for 1 h. Following incubation, the cells were washed three times with PBS to remove excess mitochondria. In all experiments, the volume of the isolated mitochondria added did not exceed 10% of the total volume.

### Cell viability tests

Cells were plated in a 96 well plate, 10,000 cells per well, in 100 ml of glucose-free medium (glucose-free DMEM, dialyzed FBS, 5 mM galactose, 1% glutamine and 1% penicillin-streptomycin). The following day, mitochondria isolated from HeLa DsRed2-mito cells were added at the indicated concentrations, and incubated with the cells for 72 h. Mitochondrial isolation buffer alone was used as control. Following 3 washes of cells with PBS and replacing with fresh medium, cell viability was assayed using CellTiter-Blue® (Promega, Madison, WI) a fluorescence-based assay, according to the manufacturer’s manual.

Cell culture medium was used as the blank. To ensure that the isolated mitochondria themselves did not influence the results, we examined isolated mitochondria without cells and found that they have negligible fluorescence.

### Complex II + III activity tests

Cells were plated in 10 cm plate in glucose-free medium. The following day mitochondria isolated from HeLa DsRed2-mito cells were added at a final concentration of 450 μg/ml, and incubated for 72 h. Complex II + III activity assays were performed as previously described^50^.

### Treatments of isolated mitochondria

All treatments were performed on ice or at 4 °C.

#### Digitonin treatment

Digitonin was added to mitochondria isolated from HeLa DsRed2-mito cells at the indicated concentrations (w/w) at a final volume of 100 μl. For control, vehicle (DMSO) was added. Following 10 min incubation, the mitochondria were re-suspended with 1.5 ml mitochondrial isolation buffer, and washed three times by centrifugation at 12,000 g for 10 min and re-suspension of the mitochondrial pellet with 1.5 ml mitochondrial isolation buffer. Mitochondrial final concentration was determined by the Bradford assay.

#### Swelling treatment

Mitochondria isolated from HeLa DsRed2-mito cells were diluted in swelling buffer (20 mM HEPES) or in mitochondrial isolation buffer as control, at a mitochondria:buffer ratio of 1:10 (v/v) for the indicated time periods, at a final volume of 300 μl, followed by three washes, re-suspension and mitochondrial protein concentration measurement as described above.

#### Trypsin treatment

Trypsin without calcium, magnesium and phenol red (Biological Industries, Beit Ha’emek, Israel) was added to mitochondria isolated from HeLa DsRed2-mito cells at the indicated concentrations (w/w) and indicted time periods, at a final volume of 100 μl. For control, FBS was added to a final concentration of 20% (v/v). Following the incubation, the mitochondria were washed as described above, and the mitochondrial concentration was re-determined.

In order to ensure that the treatments did not influence the fluorescence of the mitochondria, the fluorescence of the treated and control mitochondria was determined and found to be the same.

### Fluorescence tests and calculations

Experiments were performed in Thermo Fischer Scientific Nunclon 96 flat transparent cells plates, in a final volume of 100 μl PBS. The fluorescence was analyzed using the Infinite M200 pro plate reader (Tecan, Morrisville, NC), and Magellan software (Tecan, Morrisville, NC), with multiple reads per well. Excitation/emission wavelengths were 554/583 nm. Gain of the fluorescence test was optimized for each plate by the software. Each sample was tested in triplicates. When isolated mitochondrial fluorescence was recorded, PBS with mitochondrial isolation buffer was used as the blank. When fluorescence of cells following mitochondrial transformation was recorded, fluorescence of cells without mitochondrial transformation treatments was set up as the blank. Mitochondrial controls and treatments are described above. Calculation of relative transformation was as follows: [(treatment-blank)/(control-blank)]^*^100.

### Confocal microscopy

Cells were plated in 24 well plates on 12 mm cover-slips. The cells were fixed with 4% (v/v) paraformaldehyde for 15 min, washed three times with quenching solution (1% NH4Cl in PBS), permeabilized with 0.1% tritonX100 in PBS for 15 min, and washed three times with PBS. One drop of anti-fade mounting medium (Vectashield Burlingame, CA) was gently pipetted on the microscope slide, and after quick drying the cover-slip was moved to a microscope slide and sealed with nail polish. The slides were kept at 4 °C, viewed with an Olympus FV1000 confocal microscope (Olympus, Center Valley, PA) and analyzed using Olympus Fluoview software (Olympus).

### Immunofluorescence staining

Cells were plated in 24 well plates on 12 mm cover-slips. The cells were fixed with 4% (v/v) paraformaldehyde for 15 min, washed three times with quenching solution (1% NH4Cl in PBS), permeabilized with 0.1% tritonX 100 in PBS for 15 min, and washed three times with PBS. Blocking was done with 5% FBS in PBS for 30′ in incubator, following by staining with anti-NDUFS1 antibody (Proteintech, IL, USA) diluted 1:50 in 5% BSA in PBS for 1 h in incubator. Following 3 washes with 5% FBS in PBS, the cells were treated with 488-conjugated antibody (Jackson, PA, USA) diluted 1:200 in 5% BSA in PBS for 1 h in incubator. The cells were washed 3 times in 5% BSA in PBS, and cover-slips were prepared for confocal microscopy as described above.

### Live microscopy

Cells were grown in 8 chambers plates. Microscopy was performed inside a 37 °C and 5% CO_2_ humidified atmosphere incubator, with a Zeiss LSM 710 confocal microscope (Carl Zeiss, Oberkochen, Germany). Images were taken every 5 min, and were analyzed by ZEN software (Carl Zeiss, Oberkochen, Germany).

### TEM analysis

Cells were plated on 8 chambers plates as described above. Cells were fixed in a solution containing 2% paraformaldehyde, 2.5% gluteraldehyde (EM grade), in 0.1 M sodium cacodylate buffer, pH 7.3 for 2 h at R.T., followed by 24 h at 4 °C. Cells were washed 4 times with sodium cacodylate and post-fixed for 1 h with 1% osmium tetroxide, 1.5% potassium ferricyanide in sodium cacodylate, and washed 4 times with the same buffer, followed by dehydration with a graded series of ethanol solutions (30, 50, 70, 80, 90, 95%) for 10 min each and then 100% ethanol 3 times for 20 min each, followed by 2 changes of propylene oxide. Cells were then infiltrated with a series of epoxy resin (25, 50, 75, 100%) 24 h each and polymerized in the oven at 60 °C for 48 h. The blocks were sectioned by an ultramicrotome (Ultracut E, Riechert-Jung) and sections of 80 nm were obtained and stained with uranyl acetate and lead citrate. Sections were examined by Jeol 1400 Plus transmission electron microscope, with an Orius Gatan CCD camera and the Gatan digital micrograph program. TEM analysis was performed by the electron microscopy service laboratory (EML) of The Hebrew University, Faculty of Medicine, Jerusalem, Israel.

### Inhibition of mitochondrial transformation

HepG2 cells or healthy fibroblasts were plated on 96 well plates as described above, and the following day inhibition experiments were performed.

#### Macropinocytosis inhibition

Cells were pre-treated with amiloride (Sigma, catalog number A7410), 5-(N-ethyl-N-isopropyl)-Amiloride (EIPA; Cayman chemicals, MI, USA, catalog number 14406) or vehicle (DMSO) at the indicated concentrations for 30 minutes, and then DsRed2-mito isolated mitochondria were added (300 μg/ml) for 1 h incubation^51^,^52^.

#### Clathrin-mediated endocytosis inhibition

Three different treatments were used in order to inhibit clathrin-mediated endocytosis^51^,^53^: (1) Hypertonic sucrose treatment was done by adding sucrose or vehicle (cell medium) to final concentration of 0.5 M for 30 minutes before mitochondria were added. (2) Cytosolic acidification was done by adding ammonium chloride (25 mM) or vehicle (cell medium) for 30 minutes before mitochondria were added. (3) For potassium depletion treatment, cells were washed twice with potassium depletion buffer (140 mM NaCl, 20 mM HEPES, 1 mM CaCl2, 1 mM MgCl2, 1 mg/ml d-glucose, pH 7.4) or control buffer (140 mM NaCl, 20 mM HEPES, 1 mM CaCl2, 1 mM MgCl2, 1 mg/ml d-glucose, 10 mM KCl, pH 7.4), incubated 15 minutes in hypotonic potassium depletion buffer (potassium depletion buffer:H2O 1:1) or hypotonic potassium depletion control buffer (control buffer:H2O 1:1) and washed twice in potassium depletion buffer or hypotonic potassium depletion control buffer, which were also the incubation buffers. Following the treatments, DsRed2-mito isolated mitochondria were added (300 μg/ml) for 1 h incubation.

For both macropinocytosis inhibition and clathrin-mediated endocytosis inhibition, at the end of the incubation, excess mitochondria were removed by three washes with PBS, and membrane-bound mitochondria were removed by incubation with heparin (200 μg/ml, 30 minutes) and 3 PBS washes.

### Image analysis

Image analysis (Table S1) was done using Image-Pro 7.0 (Media Cybernetics, MD, USA), by the Light Microscopy and Imaging team of The Hebrew University Faculty of Medicine, Jerusalem, Israel.

### Statistical analysis

Statistical analysis was carried out by a one-sample t test using the GraphPad web site (http://www.graphpad.com; GraphPad Software, Inc, CA).

## Additional Information

**How to cite this article**: Kesner, E. E. *et al*. Characteristics of Mitochondrial Transformation into Human Cells. *Sci. Rep.* **6**, 26057; doi: 10.1038/srep26057 (2016).

## Supplementary Material

Supplementary Information
