# Supplementary material for: Characteristics of Mitochondrial Transformation into Human Cells
Source: Sci Rep. 2016 May 17;6:26057. doi: 10.1038/srep26057 (PMC4868981; doi:10.1038/srep26057)
Supplement: Supplementary Information [file srep26057-s1.pdf]

Characteristic Of Mitochondrial Transformation Into Human Cells

Kesner, E. E.[1], Saada-Reich, A.[2] and Lorberboum-Galski, H.[1*]

[1] Department of Biochemistry and Molecular Biology, Institute for Medical Research Israel-Canada (IMRIC), Faculty of Medicine, Hebrew University of Jerusalem, Jerusalem, 91120, Israel

[2] Monique and Jacques Roboh Department of Genetic Research, Department of Genetics and Metabolic Diseases, Hadassah, Hebrew University Medical Center, Jerusalem, Israel

Figure S1

A.

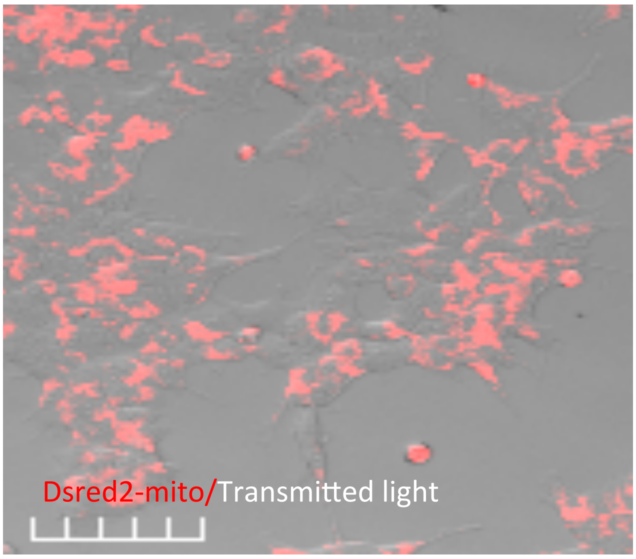

B.

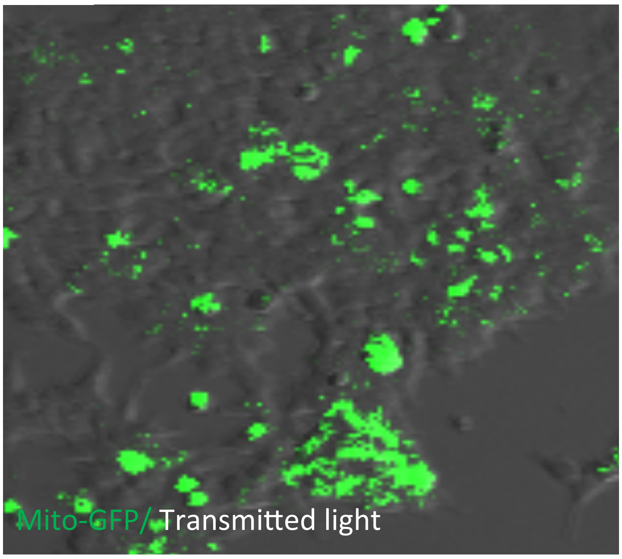

Figure S2

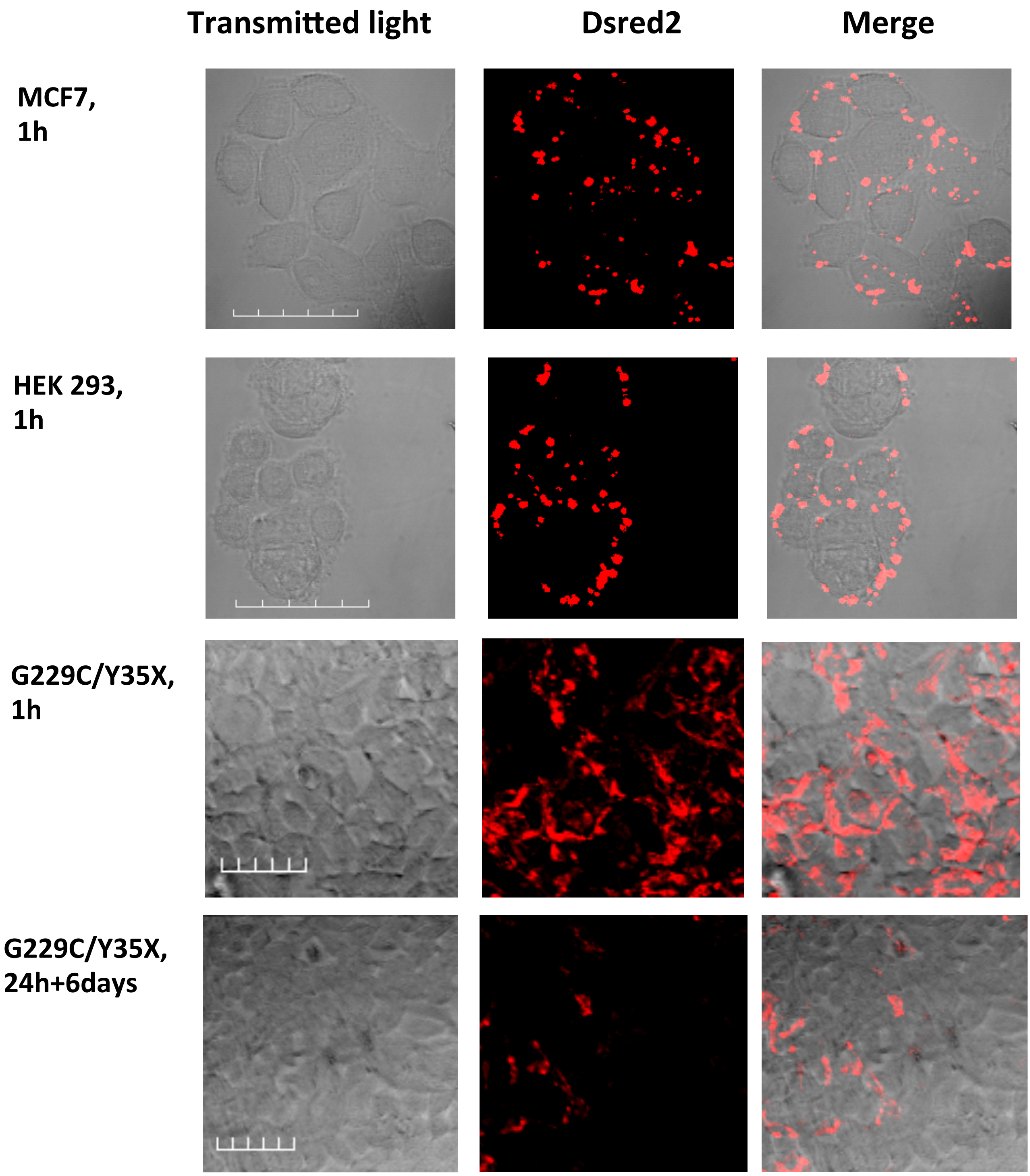
Transmitted light
Dsred2
Merge
MCF7, 1h
HEK 293, 1h
G229C/Y35X, 1h
G229C/Y35X, 24h+6days

Figure S3

A.

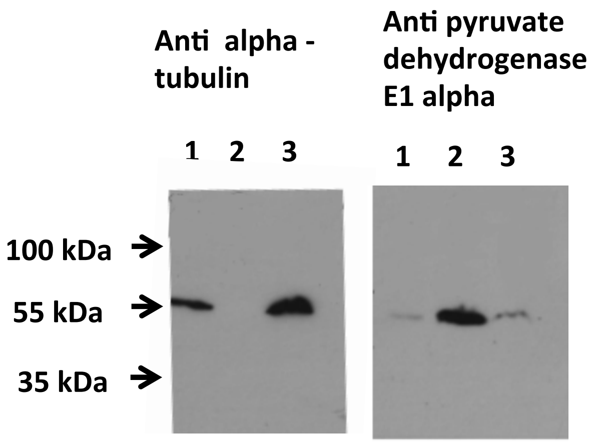
Anti alpha - tubulin
Anti pyruvate dehydrogenase E1 alpha
1 2 3
1 2 3
100 kDa
55 kDa
35 kDa

B.

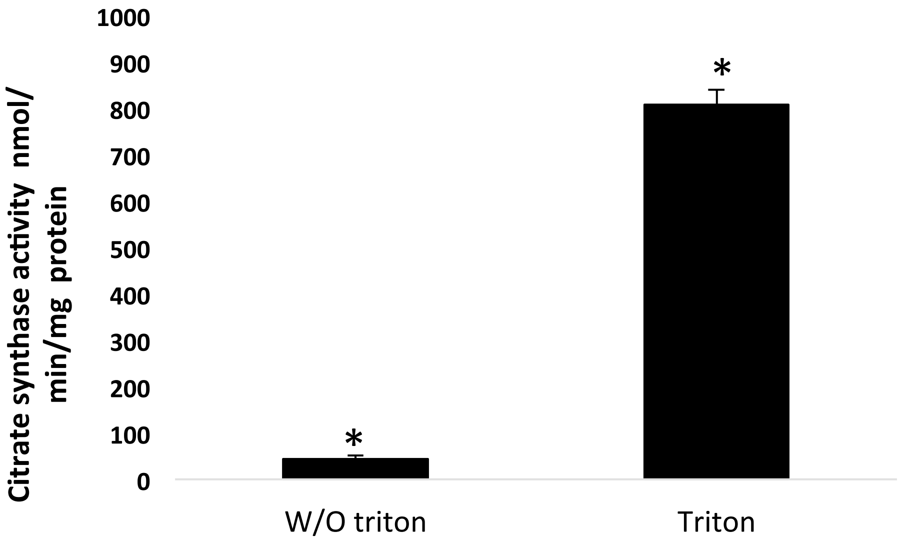
Citrate synthase activity nmol/ min/mg protein
1000
900
800
700
600
500
400
300
200
100
0
*
*
W/O triton
Triton

Figure S4

A.

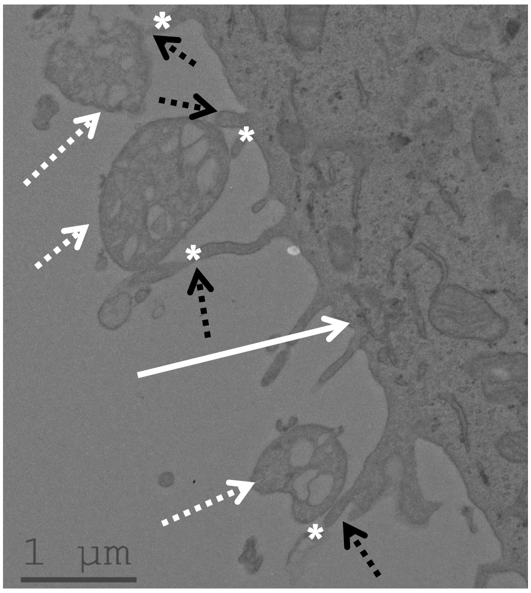

B.

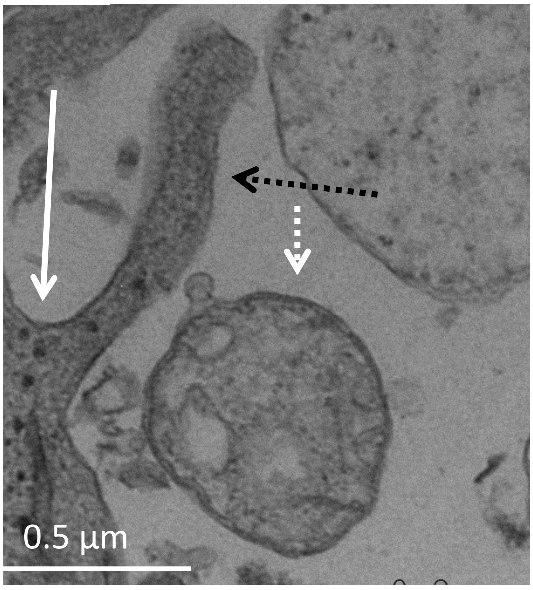

C.

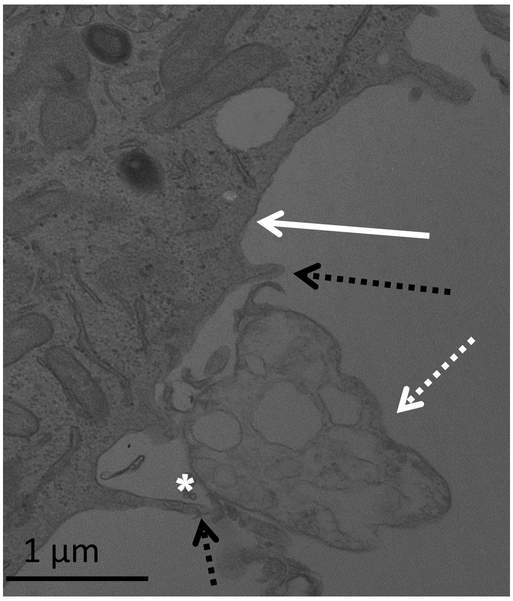

D.

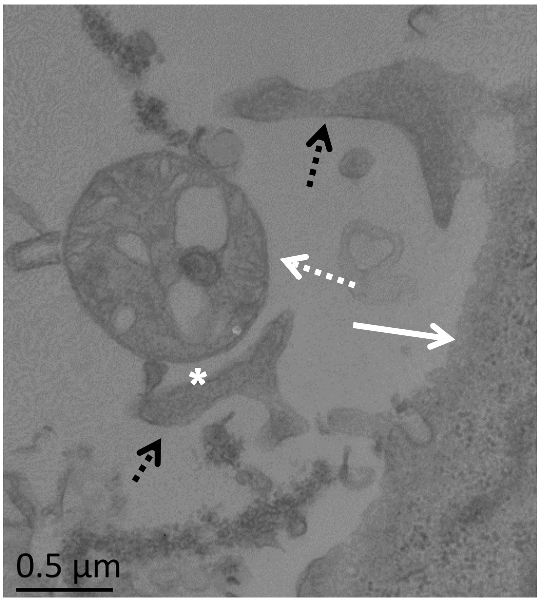

Figure S5

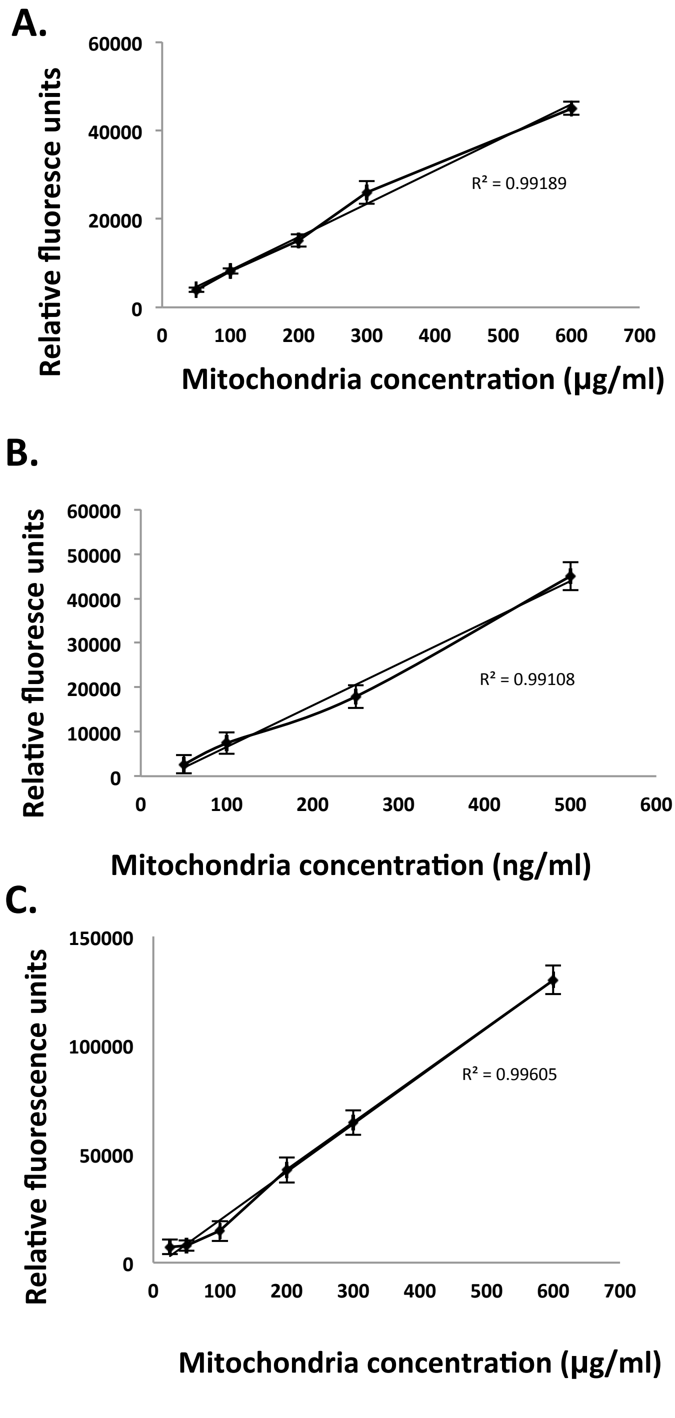
A.
Relative fluoresce units
60000
40000
20000
0
0
100
200
300
400
500
600
700
R² = 0.99189
Mitochondria concentration (µg/ml)
B.
Relative fluoresce units
60000
50000
40000
30000
20000
10000
0
0
100
200
300
400
500
600
R² = 0.99108
Mitochondria concentration (ng/ml)
C.
Relative fluorescence units
150000
100000
50000
0
0
100
200
300
400
500
600
700
R² = 0.99605
Mitochondria concentration (µg/ml)

Figure S1. (A) Transfection of HeLa cells with the plasmid DsRed2-Mito. HeLa cells were transfected with the plasmid DsRed2-Mito. Images were taken using confocal microscope (Olympus FV1000, X60). Red – Dsred2. Scale bar: 50 µm. (B) Transfection of HEK 293 cells with the plasmid CMV/myc/mito/GFP. HEK 293 cells were transfected with the plasmid CMV/myc/mito/GFP. Images were taken using confocal microscope (Olympus FV1000, X60). Green– GFP. Scale bar: 50 µm

Figure S2. (A) Transformation of isolated mitochondria into cells. Mitochondria were isolated from HeLa-DsRed2-mito cells and incubated (300 µg/ml) for 1 h or 24 h with MCF7 cells, HEK 293 cells, or fibroblasts taken from a patient, with the G229C/Y35X mutation. Following the incubation the cells were washed 3 times with PBS, and the medium was replaced with fresh medium. In cases where cells were grown for more than 24 h following the incubation, the cells were divided daily. Images were taken using a confocal microscope (Olympus FV1000; MCF7 and HEK 293: X60; G229C/Y35X fibroblasts: X20). Scale bar: 50 µm.

Figure S3. Mitochondria isolation completeness and viability verification. (A) HeLa- Dsred2-mito cells were homogenized and fractionized to (1) cytosolic fraction and (2) mitochondrial fraction, or (3) subjected to standard protein extraction of whole cellular content. Samples were taken for western blot analysis (100 µg protein/lane), using (left panel) anti α-tubulin antibody (to detect cytosolic content) and (right panel) anti pyruvate dehydrogenase E1α antibody (to detect mitochondrial content). (B) Mitochondria were isolated form HeLa-Dsred2-mito cells and were subjected to citrate synthase assay, which verifies both mitochondrial activity and completeness.
Citrate synthase activity is represented as nmol/mim/mg protein. *$P<0.05$. Error bars represent ±SEM, n=3.

Figure S4. TEM microscopy of mitochondrial interaction with recipient cells. (A-D) Mitochondria were isolated from HeLa-DsRed2-mito cells and were incubated with HepG2 cells (300 µg/ml) for 1 h. Following incubation, excess mitochondria were removed by three washes with PBS, and the cells were examined by TEM. Pictures were taken using a Jeol 1400 Plus TEM with an Orius Gatan CCD camera and Gatan digital micrograph program (A: x2500 magnification; B: x10,000 magnification; C: x3,000 magnification; D: x6,000 magnification). White arrow: HepG2 cells; white dashed arrow: exogenous mitochondria; black dashed arrow: cellular excitations; asterisk: contact site.

Figure S5. Quantitation of Florescence mitochondria and mitochondrial transformation.
(A-B) Mitochondria were isolated from HeLa-Dsred2-mito cells, diluted to the indicated concentrations in PBS, and fluorescence was recorded using a plate reader. PBS with mitochondria isolation buffer was used as blank. (A) Micrograms concentrations. (B) Nanograms concentrations. Error bars represent ±SEM. N=3.
(C) Mitochondria were isolated from HeLa-DsRed2-mito cells, and added at the indicated concentrations to HepG2 cells plated in a 96-well plate. Following 1h incubation, the cells were washed three times in PBS to remove excess mitochondria, and the fluorescence was recorded in a plate reader. Cells with PBS and mitochondria isolation buffer were used as blank. Error bars represent ±SEM. N=3.

Table S1. Red - Green co-localization parameters quantification

Pearson's correlation Rr=	0.210056
Overlap coefficient R =	0.21507
Overlap coefficient k1=	0.036534
Overlap coefficient k2=	1.266086
Co-localization (ch2>0) m1=	0.14702
Co-localization (ch1>0) m2=	0.566253

Figure 3B1 was subjected to image analysis by using Image-Pro 7.0 for quantification of green-labeled exogenous mitochondria co-localization with red-labeled endogenous mitochondria.
